# The Sensory Significance of Apocarotenoids in Wine: Importance of Carotenoid Cleavage Dioxygenase 1 (CCD1) in the Production of β-Ionone

**DOI:** 10.3390/molecules25122779

**Published:** 2020-06-16

**Authors:** John J. B. Timmins, Heinrich Kroukamp, Ian T. Paulsen, Isak S. Pretorius

**Affiliations:** 1 Centre of Excellence in Synthetic Biology, Department of Molecular Sciences, Macquarie University, Sydney, NSW 2109, Australia; john.timmins@hdr.mq.edu.au (J.J.B.T.); ian.paulsen@mq.edu.au (I.T.P.); 2 Biomolecular Discovery and Design Research Centre, Macquarie University, Sydney, NSW 2109, Australia; 3 Chancellery, Macquarie University, Sydney, NSW 2109, Australia; sakkie.pretorius@mq.edu.au

**Keywords:** apocarotenoid, carotenoid cleavage dioxygenases, β-ionone, β-damascenone, wine aroma

## Abstract

Olfactory cues are key drivers of our multisensory experiences of food and drink. For example, our perception and enjoyment of the flavour and taste of a wine is primarily steered by its aroma. Making sense of the underlying smells that drive consumer preferences is integral to product innovation as a vital source of competitive advantage in the marketplace, which explains the intense interest in the olfactory component of flavour and the sensory significance of individual compounds, such as one of the most important apocarotenoids for the bouquet of wine, β-ionone (violet and woody notes). β-Ionone is formed directly from β-carotene as a by-product of the actions of carotenoid cleavage dioxygenases (CCDs). The biological production of CCDs in microbial cell factories is one way that important aroma compounds can be generated on a large scale and with reduced costs, while retaining the ‘natural’ moniker. The CCD family includes the CCD1, CCD2, CCD4, CCD7 and CCD8; however, the functions, co-dependency and interactions of these CCDs remain to be fully elucidated. Here, we review the classification, actions and biotechnology of CCDs, particularly CCD1 and its action on β-carotene to produce the aromatic apocarotenoid β-ionone.

## 1. Multisensory Flavour Perception

**Figure 1:**
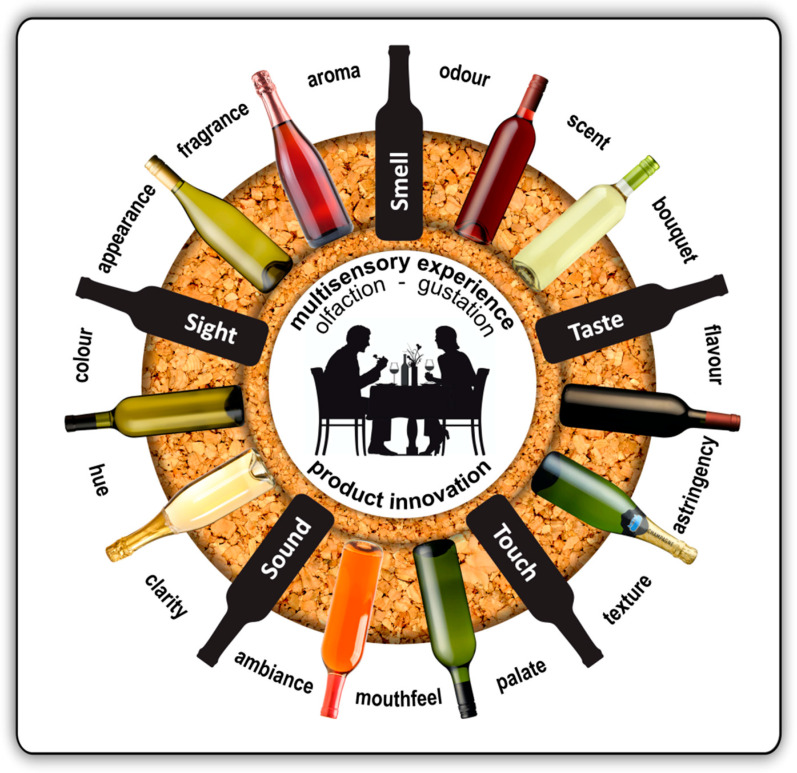
Product innovation in the wine industry is fundamentally driven by designing the appearance, aroma and flavour of wine according to the sensory preferences of consumers in target markets. The enjoyment of a wine is a multisensory experience involving all five senses, i.e. sight, smell, taste, touch and sound. Olfactory cues (smell, aroma, fragrance, odour, scent, bouquet) play a dominant role in the perception of a wine’s flavour. Consumers’ sense of flavour is built on both olfactory and gustatory (taste, palate) inputs. However, the overall experience and enjoyment of a wine’s smell and taste are augmented by what consumers see (appearance, colour, hue, clarity of the product), and what they touch (mouthfeel, texture), hear and feel (ambience, company).

The appeal and success of products in consumer markets are built on the fundamental business principle of continuous innovation. A key driver of product innovation in the food and beverage industries is the ability to tailor a product’s appearance, fragrance and flavour according to predetermined specifications aligned with consumer preferences in target markets [1]. Consumers’ multisensory perception and enjoyment of edible and potable products are built upon the interactive integration of visual (sight), olfactory (smell), gustatory (taste), textural (touch) and auditory (hearing) cues and inputs (Figure 1). However, our sense of odours plays a dominant role in our multisensory perception of flavour and enjoyment of food and drink [2,3,4]. 

**Figure 2:**
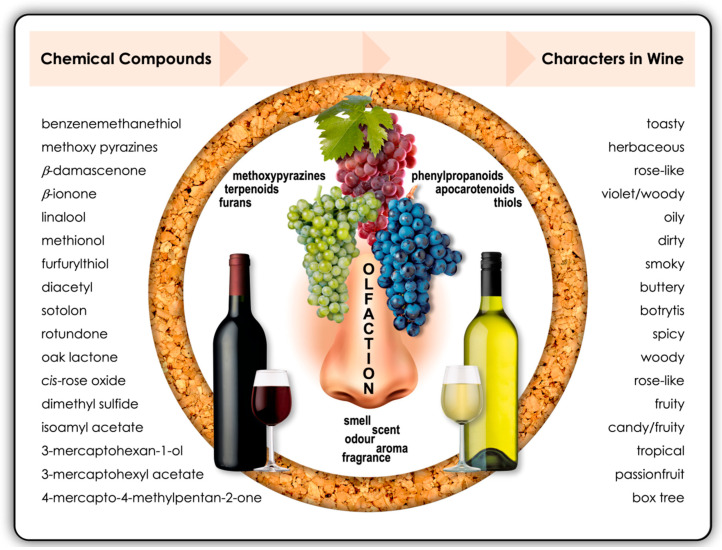
The absolute and relative concentrations of methoxypyrazines, terpenoids, furans, phenylpropanoids, thiols and apocarotenoids determine the olfactory and gustatory perception of wine. The listed chemical compounds on the left-hand side of the diagram each contribute to specific characters in wine, as indicated on the right-hand side. These impactful compounds are more prominent in certain varietal wines and styles. The objective is to achieve the correct balance of some or all of these compounds according to wine preferences in specific markets.

In the case of wine, general tasting terms used by winemakers, consumers and wine critics, such as *wine aroma* and *wine bouquet*, are neither precise nor scientific. However, such terms are useful to classify the origins of where the *nose* of a wine comes from. Usually, the term *wine aroma* refers to the ultimate combination of fragrances experienced by the wine drinker. The *wine aroma* represents the combined aromatic contributions of the grape variety (primary aroma), the fermentation process (secondary aroma) and the *wine bouquet* (or tertiary aroma). Put differently, the grape-derived compounds provide a varietal distinction in addition to giving wine its basic flavour construct, while yeast fermentation and ageing gives wine its *vinous character* [5]. For example, grape-derived floral monoterpenes largely define Muscat-related wines, while yeast fermentation generates or facilitates the release of sensorially important volatile metabolites, such as esters, higher alcohols, carbonyls, volatile fatty acids and sulfur compounds (Figure 2). 

Some aroma-active compounds like methoxypyrazines are chemically stable and are found in both grapes and wine. Methoxypyrazines are responsible for the characteristic *green*, *herbaceous* or *vegetative* aromas of Sauvignon Blanc and Cabernet Sauvignon. Other grape-derived non-volatile, flavour-inactive precursor compounds [e.g., cysteine-bound conjugates, *S*-4-(4-methylpentan-2-one)-l-cysteine and *S*-3-(hexan-1-ol)-l-cysteine (Cys-3MH)] require the enzymatic release and/or conversion by yeast to produce aroma-active volatile thiols, such as 4-mercapto-4-methylpentan-2-one (4MMP), 3-mercapto-hexanol (3MH) and 3-mercapto-hexylacetate (3MHA). For example, volatile thiols like these confer the characteristic grassy (*box tree*) and fruity (*tropical*, *passionfruit*, *guava*) notes in Sauvignon Blanc wines [5,6].

**Figure 3:**
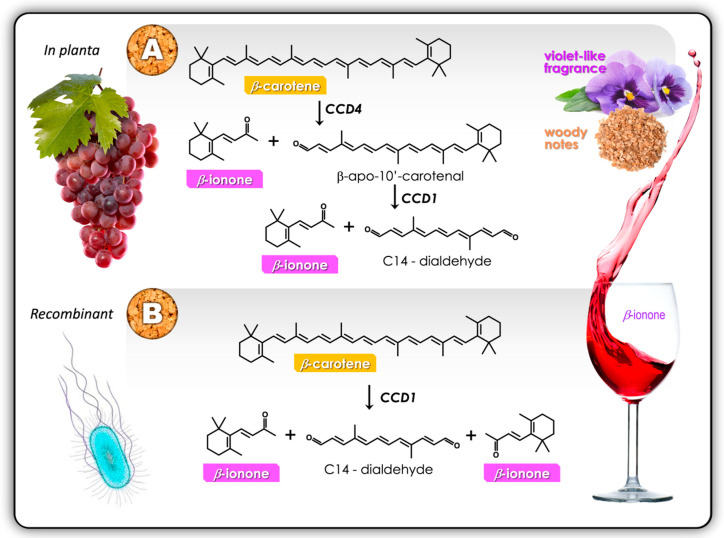
The biosynthesis of the apocarotenoid β-ionone. β-Ionone confers *violet*-like and *woody* notes to wine. (**A**) The spatio-temporal separation of enzymes and their substrates in plants results in a stepwise release of β-ionone, whereas (**B**) CCD1 has been reported to act on both ends of the β-carotene molecule simultaneously in recombinant production systems.

Apart from their capacity to produce sensorially impactful metabolites during fermentation, yeast strains also differ significantly in their ability to release and/or convert grape varietal compounds such as the aforementioned aromatic thiols [6]. The C_13_ aromatic apocarotenoid β-ionone (*violet* and *woody* notes) is a by-product of the actions of carotenoid cleavage dioxygenases (CCDs), which are expressed in grapes. β-Ionone formation from β-carotene is dependent on the presence of *VvCCD1* and *VvCCD4* (Figure 3). The other important aromatic C_13_ apocarotenoid β-damascenone (*rose*-like aroma) is derived indirectly from β-carotene via neoxanthin in wine by oxidative cleavage, followed by enzymatic reduction and acid catalysis reactions. The role of CCDs in the oxidative cleavage of neoxanthin, however, still remains unclear.

The quest to find practical ways of optimising the production of apocarotenoids and other natural aromatic compounds in wine drives much of the research into the different classes, structures and sources of enzymes, such as carotenoid cleavage dioxygenases (CCDs). Knowledge gained from such studies could offer prospects for the development of fermentation strategies (e.g., mixed-culture ferments) and wine yeast starter strains with an optimised apocarotenoid-producing capability that could assist winemakers in their effort to consistently produce wine to definable sensory specifications. 

## 2. Classification, Structure and Sources of CCDs

The history of the elucidation of the carotenoid cleavage dioxygenase (CCD) family has been spread over the last twenty-odd years and still remains to be finalised. Through recent characterisation efforts, the role of the CCD family members, namely CCD1, CCD2, CCD4, CCD7 and CCD8, is becoming clearer. The present genesis of interest in CCDs began with the perceived action of CCD1 on β-carotene to liberate β-ionone and the application of this process to the production of enhanced aromas in wine. Further studies led to the in-detail characterisation of CCD1; this ignited the interest in other CCDs and started the exploration of the question of what/where/which CCDs might be responsible for this β-carotene to β-ionone metabolic conversion and how research on this topic could, in the end, improve the flavour and taste of wine. Consequently, this paper seeks to review the history of the understanding of the functions of CCD1 and to lay a foundation for future research to clarify its true role *in planta* and its relationship with the other members of its family.

**Figure 4:**
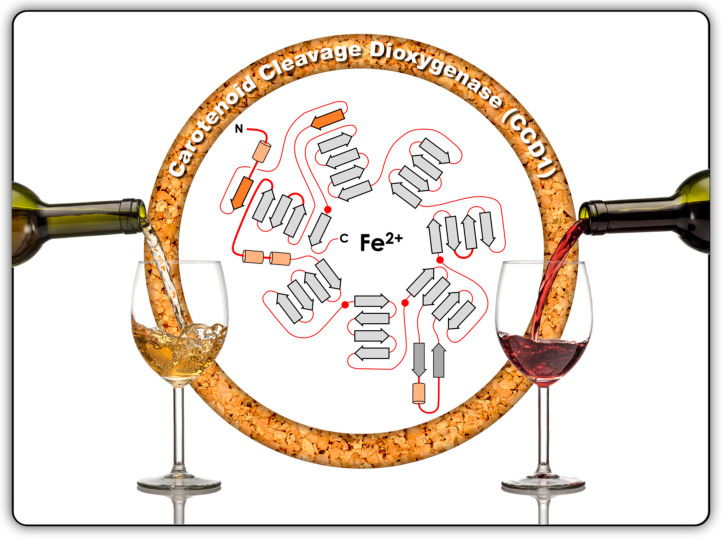
Typical structure of the carotenoid cleavage dioxygenase (CCD) family. The protein structure of the CCDs consists of seven β-sheets with a Fe^2+^ molecule at the catalytic centre of the propeller-like structure [9]. The structure contains four highly-conserved histidine molecules, which bind the Fe^2+^ molecule. While the propeller-like structure and histidine placements are conserved within the CCD family, the CCDs differ in their amino acid sequences.

The gene symbol CCD was first adopted by Steven Schwartz and colleagues, and the carotenoid cleavage dioxygenase 1, CCD1, was first characterised by the same group in 2001 [7]. This CCD1, similar to the other members of the CCD family, has a protein structure consisting of seven β-sheets forming a propeller structure (Figure 4), with a Fe^2+^ molecule at the centre for its catalytic activity [8,9,10]. This structure contains four highly-conserved histidine molecules (red dots surrounding the centre in the structure) which bind the Fe^2+^ [11], with the iron II molecule being a co-factor in the presence of oxygen for the functioning of this non-haeme enzyme [12]. While the propeller structure and histidine placements are conserved, the various CCDs differ in their amino acid sequence, with little similarity between their various clades [9].

## 3. Sources of Carotenoid Cleavage Dioxygenase 1

Table 1 contains a selected list of sources on various cyanobacterium, plant and animal CCD1s or its isoform or homologue. For a more comprehensive list of plant CCD1s, CCD4s, CCD7s and CCD8s, refer to the article by Priya & Siva [13], and for the more recently characterised CCD2, to Frusciante et al. [14], Ahrazem et al. [15] and Demurtas et al. [16]; for a phylogenic chart of CCD proteins—CCD1, CCD4, CCD7 and CCD8—refer to the article by Baba et al. [17].

Most of the characterisation work on CCD1s has been carried out in *Escherichia coli*, *Saccharomyces cerevisiae* and on a number of plants, particularly *Arabidopsis thaliana*, *Crocus sativus*, *Osmanthus fragrans* and *Vitis vinifera* [7,18,22,23,31]. For CCD4, the investigation of the functions and characterisation of the enzyme and its isoforms was firstly carried out on *Arabidopsis thaliana* and *Chrysantemum morifolium*, then on *Crocus sativus* [15,22,32] and on *Vitis vinifera* [21]. The initial characterisation of the two interlinked enzymes CCD7 and CCD8 was published by Schwartz and colleagues with work on *Arabidopsis thaliana* [33], but was followed by further experimentation and review work over several years, mainly conducted on the mycorrhizal roots of *Medicago truncatula*, by groups working in Halle, Germany [24,29,34,35,36]. More recently, CCD2 was characterised via experiments on the stigma of *Crocus sativus*, where it cleaves zeaxanthin rather than β-carotene [14,15,16]. 

The investigation into the presence and role of carotenoid cleavage enzymes *in planta* over the last several years has kept adding to the clarification and individualisation of different functions and their specific locations within the plant kingdom. This leads to the question of whether there are more such CCD classes to be discovered in the future and/or whether various combinations, e.g., CCD4 + CCD1 and CCD7 + CCD8, can be better defined and expanded, not just in plants but perhaps in other eukaryotes as well.

## 4. Biological Functions of CCDs

C_40_ carotenoids and their oxidative cleavage products, the apocarotenoids, are important compounds in nature, with the carotenoids functioning as tissue pigments and cell photoprotectants, amongst others roles, while the apocarotenoids act as signalling molecules within the organism and as attractants to insects for pollination, such as bees, or as repellents to destructive insects, such as beetles. One important apocarotenoid is β-ionone (apo-β-caroten-9-one), a low-threshold aroma product having an odour threshold of 0.007 nL L^−1^ in water [37,38,39], present in fragrant plants such as the damask rose (*Rosa damascena*) and sweet olive (*Osmanthus fragrans*), with a characteristic aroma of violet/woody/berry notes [40]. Hence, β-ionone is an important chemical for the flavour and fragrance industries and is necessary for the appreciation of the flavour and aroma of some wines [5].

Carotenoids can be broken down at non-region-specific positions by chemical, photochemical and oxidase-coupled mechanisms [41]. In the production of wine, the degradation of carotenoids can also occur via glycosylated intermediates, which are then liberated to the free aromatic aglycone through enzymatic activity and/or acid hydrolysis in the low pH environment of wine, at 3.0–3.5 [42]. However, in nature, carotenoids are enzymatically cleaved at regionally specific positions; the C_40_ β-carotene can be doubly cleaved at its 9/10, 9’/10’ carbon bonds (Figure 3) to produce two molecules of the C_13_ β-ionone plus one of C_14_ dialdehyde [23]. The enzyme responsible for this specific symmetrical activity is one belonging to the family of carotenoid cleavage dioxygenases, CCD1, although other substrates result in varying products from the cleavage at different sites by this protein [7]. CCD genes exist throughout much of the eukaryote world, mostly in the plant kingdom, but are also found in fungi and the occasional animal (Table 1). The cyanobacterium *Nostoc commune*, containing the NosCCD (which is an ortholog of plant CCD1), also has the carotenoid enzyme genes dispersed across its genome, rather than clustered, as in eubacteria [24]. The yeast *Xanthophyllomyces dendrorhous* also synthesises a number of carotenoids, including β-carotene. Mammals, such as the ferret (but not including *Homo sapiens*), have a CCD-like gene, CMO2/BCO2, which specifically cleaves β-carotene at 9’/10’. 

It has been debated over a number of years whether the cleavage of the C_40_ β-carotene to the C_13_ β-ionone might be sequential and within different compartments *in planta*, from plastid to cytosol, with the final action being that of CCD1 on an intermediate C_27_ substrate, β-apo-10’-carotenal, in the cytosol [21,24,29,43]. In fact, this stepwise cleavage pathway—‘C40 => C13 + *C27* => *C13* + *C14*’—was first proposed in the early 1990s [44] but was then generally ignored [29,36] following the in vitro demonstration of the symmetrical 9/10, 9’/10’ cleavage of β-carotene, C40 => 2C13 + C14, by CCD1 in *Escherichia coli* [7] (Figure 3).

Rubio and colleagues found that CCD4, natively located in the plastids of *Crocus sativus* stigma, was more active than CCD1 in vitro in producing β-ionone [22]. Although CCD4 is phylogenetically distinct from the CCD1 enzyme, it was shown to cleave β-carotene at the 9/10 and 9’/10’ positions when heterologously expressed in *Escherichia coli*. However, *in planta*, CCD4 or CCD7—depending on the specific plant organ—appears to act at a single cleavage point on β-carotene to produce only one molecule of β-ionone and a C_27_ moiety [29,35]. When an RNAi-mediated CCD1 gene silencing study was performed in roots, 50% of the C_13_ apocarotenoid was still produced with an accumulation of C_27_ apocarotenoids, but not the C_40_ carotenoids, indicating that C_27_ derivatives, not C_40_ molecules, were the main substrates for CCD1 [29]. It was also suggested that one type of enzyme isoform is constitutively expressed, while another might be specific for a particular plant tissue; the enzymes CCD1 and CCD4, respectively, might be considered examples of this type of evolved specialisation [22]. This proposal was also iterated by others [43] in characterising the enzymes CsCCD1 and CsZCD (zeaxanthin 7/8,7/8’ cleavage dioxygenase), also in *Crocus sativus*. Again, it is suggested that the oxidative cleavage—this time of zeaxanthin—is a stepwise process involving firstly the putative hydrophobic environment of the chromoplast, and then secondly the more hydrophilic environment of a central vacuole, and that CsCCD1 is constitutively expressed while CsZCD is expressed specifically in particular tissues, such as chromoplast style cells, and enhanced under certain conditions [43]. A similar separation of the CCD enzyme function and localisation was demonstrated by Lashbrooke and colleagues [21] for *Vitis vinifera*, with VvCCD1 expressed constitutively, whereas their work on the identification and functional characterisation of VvCCD4a and VvCCD4b showed the specialised expression and catalysis of carotenoids in the plastids of leaves (VvCCD4a) and berries (VvCCD4b). 

Glycosylation has been shown to occur as part of the natural process in carotenoid metabolism [35,45]; such glycosylation has been best described in the pathway for the production of saffron, safranal and picrocrocin in *Crocus sativus*. An analogy can be drawn between the metabolism of β-carotene by CCD4 and CCD1 in various plants and the modifications of carotenoids in *Crocus sativus* where the apocarotenoid crocetin is glycosylated with between 6 to 14 units of glucose to produce several types of crocins, with crocetin located in the chromoplast while the now hydrophilic crocins accumulate in vacuoles [46]. Researchers also refer to apocarotenoid glycosylation, following the cleavage of β-carotene by CCD4, as a means to enhance the pathway flux of products and to prevent the accumulation of potentially toxic carotenoids in plastids/chloroplasts/chromoplasts by increasing the flow of such glycosylated apocarotenoids into the hydrophilic cytosol [45]. Other researchers also identified the C_27_ apocarotenoid as a subject for glycosylation with two hexose molecules, such modifications usually taking place in the cytosol where glycosylation enzymes reside [35]. No glycosidic precursors of β-ionone were found (due to the absence of a cyclohexyl 3-OH)—maintaining its concentration before and after hydrolysis—in grape aroma studies of free and bound fractions of the terpenes C_13_ apocarotenoids and C_6_ compounds [47]. However, another research group had earlier suggested that C_13_ derivatives (containing a cyclohexyl 3-OH) were glycosylated and deposited in the plant cell vacuoles [29]. A compromise between glycosylation in the plastid versus glycosylation in the cytosol was suggested. This compromise proposed that plastidial apocarotenoids, while passing through the plastidial membrane to the cytosol, would be glycosylated in plastidial vesicles [24]. Nevertheless, recent research into plant apocarotenoid transmembrane transporters indicates that not enough studies of the mechanisms for such transportation through biological membranes have been undertaken [48].

Furthermore, the results from recent experiments indicated that, *in planta*, the substrate for CCD1 was an apocarotenoid rather than the carotenoid, and it was suggested that, in fact, CCD1s could perform more of a scavenging role for cytosolic apocarotenoids [49], whereas CCD4s (leaves and berries) or CCD7s (roots and stems) should be regarded as the primary cleavage enzymes for carotenoids, as they are co-located with their substrates in plastids [35,49], and for delivering the C_27_ intermediate substrate for the action by CCD1 in the cytosol [36].

## 5. *In Planta* Conclusions for CCD1

A hypothetical pathway of β-carotene degradation *in planta* to produce β-ionone from two carotene cleavage dioxygenase groups acting in sequence is now proposed in this paper: 

(A) CCD4 (in leaves and berries) or CCD7 (in roots and stem) acting in plastids, followed by CCD1 acting in the cytosol: (i) C_40_ β-carotene is synthesised in the plastid via the MEP pathway [50]; (ii) in the lipophilic environment of the plastid, CCD4/CCD7 acts at one end of the β-carotene molecule to produce the C_13_ β-ionone and a C_27_ apocarotenoid [29,35]; (iii) an efflux pump for the transportation of C_27_ apocarotenoids from the plastid through the plastidial membrane into the cytosol, to prevent the build-up of potentially toxic β-carotene or its precursors in this compartment (unless it has the capacity to sequester the carotenoid) by shifting the metabolic equation to the right and allowing carotenoid homeostasis. 

(B) This detoxifying process might be aided by the glycosylation of the metabolic cleavage products, if they contain a 3-hydroxyl group on the cyclohexyl ring or a terminal hydroxyl group on an aliphatic chain; this glycosylation step may occur after several such modifications, and at any or all of the following locations, based on the anatomy of the plant, e.g., root, stem, leaf, style/stigma or other parts of the flower, or berry: (i) in the plastid [45,46], with modifications to the apocarotenoids via ‘metabolons’, plastid-localised multienzyme complexes [51], as a pre-cursor to, or part of, the actual glycosylation step; (ii) at the plastidial membrane in plastidial vesicles [24] or at other membrane interfaces, e.g., the cytoplasmic membrane or the cytoskeleton [16]; (iii) in the cytosol [35].

(C) This glycosylated or pre-glycosylated moiety then passes from the hydrophobic environment of the chloroplast, chromoplast and plastid through its membrane to the hydrophilic cytosol or then, sometimes, into hydrophilic vacuoles, which may provide a ubiquitous manner of storing glycosylated apocarotenoids [24]. Once in the cytosol where the enzyme CCD1 resides, the other end of the C_27_ β-carotene metabolite is cleaved at the 9/10 position to liberate another molecule of β-ionone [21,24,29], leaving a C_14_ dialdehyde metabolite in the final step of the pathway C40 => C13 + *C27 => C13 + C14* [44].

## 6. Biotechnology of CCDs

Natural products have been used over millennia as the source of important aroma compounds, but their extraction from harvested and processed flowers, leaves or roots is time-consuming and expensive. More recently, particularly in the second half of the twentieth century, synthetic compounds have been used as replacements for the natural fragrance extracts but are burdened with the tag of being ‘unnatural’ and eschewed by various groups in society, while their processing steps can, at times, be difficult and still expensive. The biological production of flavours and fragrances using CCDs, expressed from heterologous genes integrated into transformed yeast, is one way that important aroma compounds can be generated on a large scale and with reduced costs, while being able to retain the ‘natural’ tag. 

A number of the aromatic apocarotenoids produced by the actions of CCDs, especially the low odour detection threshold C_13_ metabolite β-ionone, have been appreciated by the flavour and fragrance industry for many years. But their worth reaches beyond creating exotic perfumes or expensive spices to the production of vitamins, hormones and other important chemicals, and to flavouring foods and providing beverages with a lift in their aroma profile. The volatile apocarotenoid compounds, which may only be in low concentrations in plants, such as the European noble grape varieties of *Vitis vinifera*, are already important to the wine industry for the appreciation of the flavour and aroma of some wines [52] and in determining differences in the soil, season and region of such wines [38]. Important sensory compounds in wine can vary from soil to soil, from season to season, with fluctuations in temperature, precipitation and microbiomes, and of course from one country to another and indeed from one region to another, which the French might describe as differences in *terroir* (recently reviewed by Pretorius [53]). Therefore, by enhancing the organoleptic profile of a product in a more consistent way through the available biotechnological tools, an opportunity might exist to provide advantages for the winemaker and benefits to the wine consumer.

**Figure 5:**
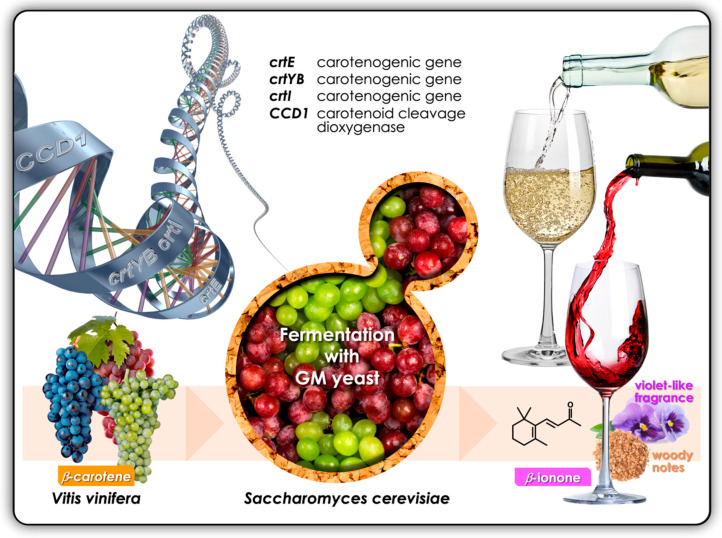
The development of ‘yeast cell factories’ for the production of the apocarotenoid, β-ionone. One approach would be to incorporate and express three heterologous carotenogenic genes (*crtE*, *crtYB* and *crtI*) and the *CCD1* carotenoid cleavage dioxygenase gene in *Saccharomyces cerevisiae*.

The benefit of using biotechnology by employing CCDs in the wine industry to enhance the fermentation products of the grapevine with desirable organoleptic properties is worth considerable attention from winemakers [54]. These CCD enzymes, such as CCD1 and CCD4, while not occurring naturally in fermentative yeasts, such as *Saccharomyces cerevisiae*, can have their genes heterologously incorporated into the yeast through genetic engineering, either by integration into the genome or by having the yeast transformed with a plasmid containing the gene. The CCD enzymes can then be released during the winemaking process to act on natural ingredients, such as β-carotene, in the crushed grape berries in the winery, to release aromatic apocarotenoids in the ensuing wine; bioengineering experiments in the laboratory would need to produce the CCD enzyme, together with a possible heterologous source of β-carotene, in order to generate β-ionone (Figure 5). Early experiments in *Escherichia coli* with the co-expression of heterologous *Erwinia herbicola* carotenoid enzyme genes and CCD1 from *Arabidopsis thaliana* yielded β-ionone from β-carotene, amongst others, which were identified through a combination of HPLC, UV-visible spectroscopy and mass spectrometry [7]. Inspired by these results in *Escherichia coli*, researchers mimicked the co-expression process in yeast, producing β-ionone from glucose [40]. This time, the carotenoid synthetic enzyme genes from *Xanthophyllomyces dendrorhous* were cloned into *S. cerevisiae*, together with the *RiCCD1* gene from raspberry, and this combination resulted in detectable levels of β-ionone. 

Later on, a modified approach to produce β-ionone at higher levels with yeast was carried out. This entailed a combination of gene deletions and other flux diverting genetic modifications to prevent pathway bottlenecks, to extend the MVA pathway of the yeast to deliver an enhanced production of β-carotene; the overexpression of the *CCD1* gene, this time using *PhCCD1* from petunia rather than raspberry, was added to the carotenoid enzyme genes to cleave the produced carotenoid substrate to produce β-ionone. The end result of this constructed yeast platform resulted in high levels of β-ionone production, especially in batch fermentations, producing 1 mg/g DCW at 50 h [55]. Continuous improvements since then have been made to this process, including the use of other yeasts than *Saccharomyces cerevisiae*, to increase the titres of β-ionone produced up to 380 mg/L in a bio-fermenter [30].

While opinions on the consumption of genetically modified organisms (GMOs) in foods remain divergent, particularly between Europe and North America, such use in wine could be problematic on a global basis. However, GMO foodstuffs are accepted and used in various countries to improve yields, e.g., canola in the USA, Canada and Australia; in fact, there is scientific consensus that, compared to conventionally-treated food, no greater harm to human health has become evident through the use of approved GMO food crops [56]. Furthermore, many drugs and vaccines are produced by biotechnological methods employing GMO processes, typified by insulin [57], and these do not seem to raise the same objections to those of improved foods being presented to the consumer via a similar technology. Perhaps by the time a GMO wine yeast expressing CCD1 is ready for the market, education of the public may make it more acceptable than the historic first GMO wine yeast, ML01 (which added a bacterial malolactic fermentation capability), turned out to be.

Wine is a complex mixture of many chemicals, mainly water, ethanol and organic acids, such as tartaric acid, but it contains small quantities of at least 71 volatile components [58], all of which contribute both to the bouquet and mouthfeel of the wine. A chemical aroma footprint for wines can be established through the combination of an instrumental analysis of the compounds together with aroma sensory data, in order to understand aroma properties [58]. The two previously mentioned apocarotenoids, β-ionone and β-damascenone, are not alone in establishing this footprint, but are representative of a panoply of such aroma compounds. In fact, there are many such molecules, derived either directly or indirectly from the grape, from the fermentation process or from the metabolic by-products of the *Saccharomyces* and non-*Saccharomyces* yeasts and malolactic bacteria themselves, which contribute to the fermentation process of the grape sugars to ethanol [54,59]. Not only can these aroma compounds display their own particular aroma characteristics, but they may also interact with each other to alter the perceptions of their primary organoleptic properties in the wine taster, producing a more complex bouquet. Adding desirable flavour compounds to wine is illegal and goes against the deeply engrained ethos of an archetypal traditional industry that values authenticity. However, there is an active discourse within the wine sector regarding the use of flavour-enhancing yeasts, such as strains producing higher concentrations of apocarotenoids through the actions of heterologously expressed CCD1. Alternatively, the application of non-GM flavour-active yeasts or combinations of natural yeasts with a capacity to produce optimal apocarotenoid levels could be developed for the increased satisfaction and enjoyment of the consumer [60]. 

## 7. A Taste of the Future

It is reasonable to expect that the importance of bioflavours will grow in the years to come. With the advent of DNA-writing and DNA-editing technologies in the emerging field of synthetic biology, it is also reasonable to expect that the possibilities for the inventive design of new-to-nature flavour-active biomolecules will expand tremendously. Such novel aroma- and flavour-enhancing biomolecules are likely to be produced with semisynthetic microbial cell factories. 

It is easy to imagine semisynthetic yeasts capable of producing aroma-enhancing compounds for the bioflavours industry. Such yeasts might even be harnessed in the food and beverage industries. A future scenario can be imagined where wine yeast strains are equipped with designer genomes to safely and consistently produce high-quality wine according to the preferences of consumers in a range of markets [54,59,60,61]. The science and technologies enabling such inventions are already available. However, the oppositional sentiments against the application of such technologies in the food and beverage industries should not be underestimated. The use of semisynthetic bioflavour-producing microorganisms in commercial winemaking is not imminent. For the time being, semisynthetic yeasts will be used as model organisms in laboratories to unravel the biosynthetic pathways of aroma and flavour compounds. 

## Figures and Tables

**Table 1:** Review of papers referencing carotenoid cleavage dioxygenase 1, its isoform or homologue.

Scientific Name	Gene	Common Name	Cited Authors
*Arabidopsis thaliana*	AtCCD1	Thale cress	[12,13,18,19,20,21,22,23,24]
*Averrhoa carambola*	AcCCD1	Star fruit	[18,19,20]
*Boronia megastigma*	BmCCD1	Brown boronia	[23,24,25,26] *
*Buddleja davidii*	BdCCD1	Summer lilac	[27]
*Citrus limon*	CitCCD1	Lemon	[12,13,19,20]
*Coffea arabica*	CaCCD1	Coffee	[12,13,19,20,24]
*Crocus sativus*	CsCCD1	Crocus	[12,13,18,22,23,24]
*Cucumis melo*	CmCCD1	Melon	[12,13,19,20,21,22,24]
*Fragaria* x *ananassa*	FaCCD1	Strawberry	[13,19,20,21,24]
*Mus musculus*	MmBCO2	Mouse	[11,28]
*Mustela putorius furo*	MpCMO2/BCO2	Ferret	[11,24]
*Nostoc commune*/spp.	NosCCD/NosNSC1	Cyanobacterium	[24,28,29]
*Osmanthus fragrans*	OfCCD1	Sweet olive	[13,19,20,21,23,24,25,30]
*Petunia* hybrid	PhCCD1	Petunia	[13,18,19,20,21,22,23,24,30]
*Phaseolus vulgaris*	PvCCD1	Common bean	[13,23]
*Prunus persica*	PpCCD1	Nectarine	[18,22]
*Rubus idaeus*	RiCCD1	Raspberry	[19,20,24]
*Rosa* x *damascena*	RdCCD1	Damask rose	[13,19,20,21,24]
*Scutellaria baicalensis*	SbCCD1	Skullcap	[27]
*Solanum lycopersicum*	SlCCD1	Tomato	[12,13,18,19,20,21,22,23,24]
*Vitis vinifera*	VviCCD1	Grape	[12,13,18,19,20,21,22,24,27,30]
*Zea mays*	ZmCCD1	Corn	[12,13,21,24]

* Relates to cleavage of β-carotene to produce β-ionone, without specifying CCD1, being pre-2001 before its Schwartz classification in [25].
